# The Association Between Mobile Game Addiction and Depression, Social Anxiety, and Loneliness

**DOI:** 10.3389/fpubh.2019.00247

**Published:** 2019-09-06

**Authors:** Jin-Liang Wang, Jia-Rong Sheng, Hai-Zhen Wang

**Affiliations:** ^1^Center for Mental Health Education, School of Psychology, Southwest University, Chongqing, China; ^2^Chongqing Youth and Vocational Technical College, Chongqing, China

**Keywords:** mobile game addiction, social anxiety, depression, loneliness, adolescents

## Abstract

As a new type of addictive behaviors and distinct from traditional internet game addiction on desktop computers, mobile game addiction has attracted researchers' attention due to its possible negative effects on mental health issues. However, very few studies have particularly examined the relationship between mobile game addiction and mental health outcomes, due to a lack of specified instrument for measuring this new type of behavioral addiction. In this study, we examined the relationship between mobile game addition and social anxiety, depression, and loneliness among adolescents. We found that mobile game addiction was positively associated with social anxiety, depression, and loneliness. A further analysis on gender difference in the paths from mobile game addiction to these mental health outcomes was examined, and results revealed that male adolescents tend to report more social anxiety when they use mobile game addictively. We also discussed limitations and implications for mental health practice.

## Background

With the fast development of mobile technology, many functions of desktop computers have been transferred to mobile devices like ipad and smartphone, which is especially the case for game applications. Mobile video games refer to games played by either single or multi players via online mobile devices. These games are particularly popular when they can be downloaded for free (e.g., “freemium games,” which are free but customers pay for extra features) (1). The latest China Internet Network Information Center's (CNNIC) report revealed that the growth rate of mobile online game has reached 9.6% and adolescents are the main user group (2). In comparison with most segments of society, adolescents are more vulnerable to Internet-related addiction because of their psychological and developmental characteristics, the easy access to Internet with a portable device, and the positive expectation of mobile gaming (3). It has been demonstrated that video game addicts suffered poorer mental health and cognitive functioning, and increased emotional difficulties, such as enhanced depression and anxiety, as well as more social isolation (4).

Despite this, relatively few studies have examined the relationship between mobile game addiction and mental health outcomes. This is because, so far, no measurement especially designed for mobile game addiction has been developed. In literature, problematic mobile video gaming has been defined as a phenomenon in which users strongly rely on mobile games and cannot help playing them repeatedly over a comparatively long period (5). Previous studies of Internet gaming disorder (IGD) have mainly focused on traditional online gaming addiction based on a desktop computer. However, recent research has suggested that there were only moderate correlations between the different forms of Internet addiction (6). In addition, although mobile game addiction has some similarity with traditional desktop computer online game addiction, there are still obvious differences. Specifically, mobile video games are characterized by portability, immediacy, and accessibility (7), which may increase the risk for addictive behavioral patterns and, thus, more severe mental health problems.

Additionally, most prior studies have treated social anxiety, depression, and loneliness as risk factors for Internet-relevant addiction (8, 9), whereas, few studies have examined the alternative direction (10). A relevant study found that the relative risk for depression in students with Internet addiction after months was 1.5 times higher than that of non-Internet addiction participants, after controlling for potential confounding variables (gender, study burden, age, rural, or urban school). This indicated that Internet relevant addiction may also lead to depression and loneliness (11). Another reason for conducting the current study was because the relationship between playing video games and psychological adjustment during adolescence is relatively scarce, which is especially true for investigating the association between playing video games and social anxiety among adolescents (12). Therefore, an investigation on this issue can help us understand how mobile game addiction may hinder adolescents' social development and would provide some guidance for mental health education practice.

## Theoretical Framework

### Mobile Game Addiction and Depression

Internet game addiction is characterized by cognitive and emotional deficits. Previous studies have reported the co-occurrence of Internet addition and depression (13, 14). In addition, a longitudinal study found that Internet game addition/depression severity at an earlier time positively predicted the depression/Internet game addition severity at a later time, which indicated that a possible bidirectional relationship existed between online gamers' depression symptoms and addiction. People cope with their emotional distress by playing online games, but the excessive use of online games for a long time may separate individuals from real-life relationships, thus causing severer mental health problems, such as depression (15). Therefore, in this study, we would expect a positive relationship between mobile game addition and depression.

### Mobile Game Addition and Loneliness

Loneliness is defined as an unpleasant experience that derives from important deficiencies in a person's network of social relationships (16). Previous studies have consistently confirmed the connection between loneliness and online game addiction (17, 18). Furthermore, loneliness is not only the cause of online gaming addiction but also the consequence; there is a possible reciprocal relationship (19). Prior research has indicated that, although playing online games may temporarily provide an escape from the negative feelings associated with social deficiencies, excessive gaming does little to facilitate the development or maintenance of real-life relationships. Instead, the substitution for interpersonal interactions in real life may exacerbate the deterioration of existing social relationships, thereby increasing loneliness (19). Thus, we would expect a positive association between mobile game addiction and loneliness in this study.

### Social Anxiety

Social anxiety, which is the most common anxiety disorder in adolescence, is the state of tension or discomfort experienced by individuals in social situations (20). The investigation on the potential effects of mobile game addiction and adolescence social anxiety is of importance considering that approximately one third of adolescents meet the criteria for an anxiety disorder (21, 22). Some literature indicates that Internet addiction, smartphone addiction, and online game addiction were all associated with an individual's social anxiety [e.g., (23)]. Individuals with a serious tendency for online gaming addiction have significantly higher social anxiety levels than those who use online games normally. Lo et al. (24) investigated the potential effects of online games on the quality of interpersonal relationships and levels of social anxiety. The results indicated that the quality of interpersonal relationships may be undermined and the amount of social anxiety may increase when teenagers spend more time playing online games (24). In the current study, we would expect a positive association between mobile game addiction and social anxiety.

### Gender Difference

Gender has been proposed as an important factor in influencing Internet use and its outcomes regarding mental health (e.g., 8). Evidence has suggested that males have a predilection toward activities that involve explosive action and combat, while females are drawn toward activities that are more social and communication focused (25). Females received more family supervision, which may prevent them from developing Internet addiction (26). In a more recent study, female video game addicts displayed significantly more somatic difficulties than male addicts (4). They further argued that female addicts may be uniquely at risk for negative physical health outcomes and sleep disturbances (4). Significant gender difference was also revealed on the association between family function and Internet addiction among adolescence (27). Females showed more negative consequences of its maladaptive mobile phone use (28). These studies highlighted the need to explore gender differences in mobile game addition and mental health problems further.

## Methods

### Participants and Data Collection Procedure

Data of this study was from the students (*n* = 600) enrolled in the seventh, eighth, and ninth grades of a junior high school in Guizhou Province. Letters describing the project were sent home to parents with a consent form inviting participation. Children whose parents provided written informed consent and who themselves gave assent completed the questionnaire in classroom settings. Prior to answering the items, participants read information about the implications of participation and data protection. The information emphasized that participation was completely voluntary and anonymous. Excluding missing or incomplete data, 578 survey responses were collected (mean age = 15 years, *SD* = 1.05). 56.7% (*n* = 328) participants were self-identified as males.

### Measures

#### Mobile Game Addiction Scale

This scale was specially developed for the measurement of mobile game addiction and included 11 items (29). Each item was rated on a Likert-type scale from 1 = completely disagree to 5 = completely agree, with the total scores ranging from 11 to 55. A higher score indicated a severer addition tendency. This scale has shown good construct validity, with χ^2^/df = 2.835, RMSEA = 0.056, 90% CI (0.044, 0.069), SRMR = 0.037, CLI = 0.970, TLI = 0.959, the Cronbach alpha coefficient in the current study was 0.84. Sample items included: “*During the last year, have you felt miserable when you were unable to play mobile video games or played less than usual?*” and “*During the last year, have you played mobile video games so that you would not have to think about annoying things?*”

#### Depression Scale

The depression subscale from the Brief Symptom Inventory (BSI) was used to assess the depression symptoms (30). The scale contains 6 items and each item was rated on a 5-point Likert scale, ranging from 1 (not at all) to 5 (extremely serious). Higher scores indicate severe depressive symptoms. We did a measurement model analysis, and the scale showed good construct validity, with χ^2^*/df* = 1.931,RMSEA = 0.040,90% CI(0.000, 0.070),SRMR = 0.020,CFI = 0.994, TLI = 0.989. The Cronbach alpha coefficient in the current study was 0.84. Sample items included: “*You feel sad*” and “*You find everything dull*.”

#### Child Loneliness Scale

The revised version of the Child Loneliness Scale was adopted to evaluate individuals' loneliness (31). The scale contains 16 items, which were answered using a 5-point Likert scale ranging from 1 (always) to 5 (never); higher scores indicate elevated loneliness. Good construct validity was exhibited in the current sample, with χ^2^*/df* = 2.833, RMSEA = 0.056, 9 % CI(0.048, 0.065), SRMR = 0.0461, CFI = 0.940, TLI = 0.918. The Cronbach alpha coefficient in our sample was 0.86. Sample items included: “*I don't have any friends*” and “*I feel lonely*.”

#### Child Social Anxiety Scale

The modified version of the Child Social Anxiety Scale was used to assess participants' social anxiety (32). The term “children” in the original scale was changed to “classmate” in the current version. The scale contains 10 items and each item was rated using a 3-point Likert scale, ranging from 1 = never to 3 = always. Higher scores indicate higher levels of social anxiety. The scale has been proved to have good construct validity in the current study, with χ^2^/*df* = 2.872, RMSEA = 0.057, 90% CI(0.044, 0.071), SRMR = 0.041, CFI = 0.951, TLI = 0.931, and the Cronbach alpha coefficient in our sample was 0.80. Sample items included: “*I think my classmates make fun of me*” and “*I'm afraid other students won't like me*.”

## Results

### Descriptive Statistics and Zero-Order Correlations Among the Study Variables

Table 1 shows the descriptive results and zero-order correlations among the study variables. Mobile addiction was positively correlated with depression, loneliness, and social anxiety, with the correlations ranging from 0.18 to 0.46 (*p*_*s*_ < 0.01).

**Table 1:** Descriptive results and zero-order correlations among the study variables.

	**Mean**	**SD**	**Addiction**	**Depression**	**Loneliness**	**Anxiety**
Addiction	27.35	8.78		0.31^**^	0.21^**^	0.25^**^
Depression	13.04	5.06	0.31^**^		0.46^**^	0.37^**^
Loneliness	34.56	9.58	0.21^**^	0.46^**^		0.44^**^
Anxiety	18.51	3.80	0.25^**^	0.37^**^	0.44^**^	

** *P < 0.01*.

### Structural Equation Modeling on the Relationship Between Mobile Game Addiction, Depression, Social Anxiety, and Loneliness

Using Amos 22.0, we conducted a structural equation analysis to examine the association between mobile game addiction, depression, social anxiety, and loneliness.

Several underlying statistical assumptions for multiple regression analysis were examined before running the structural modeling. The assumption of homoscedasticity was checked using the Levene's Test for Equality of Variances (33). The test ensured no significant differences in the variance of the three dependent variables of social anxiety, depression, and loneliness across groups defined by mobile gaming addiction (*p* > 0.05 for all cases). Thus, the assumption of homoscedasticity was not violated (34). Second, the skewness values for all variables ranged from 0.25 to 0.82 and the kurtosis values ranged from 0.27 to 0.30, which are within the acceptable range of −1 to +1 for normality (35). Thus, the violation of the normality assumption was not present in the sample data. Thirdly, the assumption of independence of residuals was confirmed by the calculation of the Durbin–Watson statistics for the dependent variables of depression (= 1.36), social anxiety (= 1.76), and loneliness (= 1.71), which are within the acceptable range of 1.5–2.5 for independence (36). Lastly, multi-collinearity was evaluated through the assessment of zero-order correlations among selected measured constructs, as calculated in Table 1. Harris and Hagger (37) noted that multicolline arity is not a serious issue if none of the correlation coefficients between variables exceeds 0.70. It is apparent that pair-wise bivariate associations between the study variables were not highly correlated with each other. Accordingly, multi-collinearity was dismissed from being a major concern in the present study (38). To conclude, the sample data were judged to meet the criteria for further analysis.

**Figure 1:**
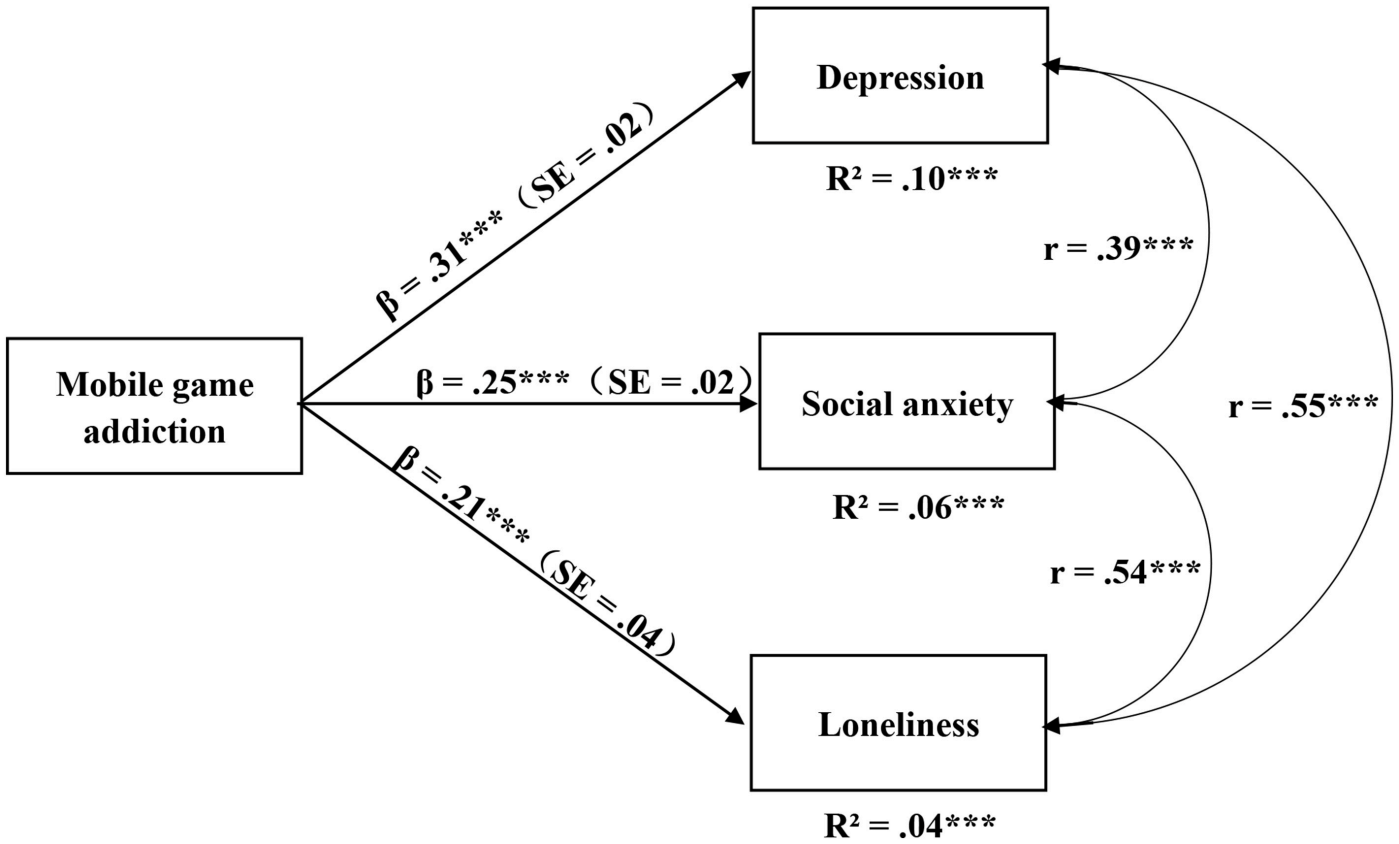
The Structural Modeling on the relation between mobile game addiction and depression, social anxiety, and loneliness. ^***^p < 0.01.

Model fit was assessed by considering multiple criteria: a Chi-square/df < 5 a root mean square error of approximation (RMSEA) of <0.08 and a comparative fit index (CFI) and a Tucker-Lewis index (TLI) of >0.90 (39). The model fit was considered acceptable when most abovementioned criteria were satisfied. Our results showed that the model fit to the data well, with χ^2^*/df* = 3.475, RMSEA = 0.065, 90% CI (0.06, 0.07), CLI = 0.937, TLI = 0.921. Mobile game addiction can explain 10% variance of depression, 6% variance of social anxiety, and 4% variance of loneliness. The standardized beta coefficients are shown in Figure 1. Mobile game addiction was positively related to depression, social anxiety, and loneliness, with β = 0.31, *p* < 0.001, β = 0.25, *p* < 0.001, and β = 0.21, *p* < 0.001, respectively.

Considering that gender was proposed as a variable that may moderate the relationship between mobile game addiction and mental health outcomes, the moderating effect of gender was tested. We created a multi-group model in AMOS to test the differences between males and females on the paths between mobile game addiction and its outcomes. The results show that significant gender differences exist considering the relationship between mobile game addiction and social anxiety. Male adolescents who used mobile game additively reported higher levels of social anxiety (β = 0.118, *p* < 0.001), depression (β = 0.280, *p* < 0.001), and loneliness (β = 0.311, *p* < 0.001), compared with female adolescents (β = 0.077, *p* < 0.001; β = 0.17, *p* < 0.01; and β = 0.16, *p* < 0.05, respectively; see Table 2 for details).

**Table 2:** Multi-group (male and female) analysis on the relationship among mobile game addiction and depression, social anxiety, and loneliness.

**Paths**	**Estimate Male**	***p***	**Estimate female**	***p***	**z-score**
Addiction→ depression	0.118	0.000	0.077	0.000	− 1.353^***^
Addiction→ anxiety	0.280	0.000	0.170	0.002	−1.411^***^
Addiction→ loneliness	0.311	0.000	0.160	0.027	−1.540^**^

** *p < 0.05*,

*** *p < 0.01*.

## Discussion

The goal of this study was to examine the associations between mobile game addiction and depression, loneliness, social anxiety, and the potential gender difference in these associations were also investigated. The results revealed that adolescent with mobile game addiction had higher self-reported depression, social anxiety and loneliness, which have supported our three hypotheses regarding the association between mobile game addiction and depression, social anxiety, and loneliness. Further, gender difference was observed in the path between mobile game addiction and social anxiety, with male adolescents having a stronger association between mobile game addiction and social anxiety. This indicates that male adolescents may experience more social anxieties if they use mobile game addictively, compared with female adolescents.

As we expected, mobile game addiction was positively associated with depression, anxiety, and loneliness, which have supported all of our three hypotheses and are in line with prior findings. Literature has consistently shown that video game addicts reported more anxiety, depression, lower positive affect and psychological well-being. Literature has also shown that Internet addictions are related to poorer emotional health, in particular depression and anxiety (40, 41). For instance, Whang et al. (41) found a significant association between degree of Internet addiction and loneliness and depression. Adolescents with high Internet use exhibited more psychopathology, as revealed by the Brief Symptoms Inventory (BSI, a reduced version of the Symptoms Checklist, SCL-90) compared with those with low those use (42). In a recent study, (4) reported that young adults addicted to video games showed increased depression and anxiety, and felt more socially isolated. The link between mobile game addiction and mental health may be due to the social isolation resulting from spending too much time gaming, which in turn leads to undermined psychological well-being (43). Our results regarding the association between mobile game addiction and loneliness are also in line with the displacement hypothesis in terms of Internet use, which argues that digital device users have spent most time in online settings, rather than offline, and their existing relationships have suffered as a result (44).

We also expected a gender difference considering the association between mobile game addiction and mental health outcomes. We found that males who were addicted to mobile games tended to suffer more social anxiety, loneliness, and social anxiety, compared with females. This finding is line with prior research (e.g., 24). Gender difference on social anxiety and loneliness has been widely reported in literature. Compared with female adolescents, male adolescents tended to lack social skills, were more socially withdrawn and disclosed less about themselves in offline communication settings (45). This is also a reason why males are more likely to be attracted to a virtual world like computer games since the online world is more comfortable and can offer more sense of security (46). This would further lead them to be more social isolated and experience more social anxiety, loneliness, and depression due to the lack of social bond in offline settings.

## Limitations and future directions

The results of this study should be viewed in light of its limitations. First, this study is a cross-sectional design. Thus, we could not determine a causal link between study variables. Future investigations should adopt an experimental design to establish the causal relationship between variables, or a longitudinal design to examine the prospective relationship among the variables. As prior studies indicated, the association between mobile game addiction and mental health problems might be reciprocal. Second, the sample is a homogeneous group of students from a middle school in China. Whether the results can be generalized to all adolescents is a question for future research.

Despite the limitations, our study has examined the association between mobile game addiction and depression, social anxiety, and loneliness, based on an adolescent sample. The results indicated that mobile game addiction was positively related to these mental health problems, and this is especially true for male adolescents as they are more likely to experience a higher level of social anxiety, depression, and loneliness after excessive use of mobile gaming. Therefore, mental health educators and practicers should be aware of the negative effects caused by addictive mobile gaming, as this is such a common phenomenon today. Specifically, attention should be given to male adolescents who are addicted to mobile gaming, as they may suffer more social anxiety.

## Data Availability

The datasets generated for this study are available on request to the corresponding author.

## Ethics Statement

The studies involving human participants were reviewed and approved by Southwest University's Human Research Ethics Committee. Written informed consent to participate in this study was provided by the participants' legal guardian/next of kin.

## Author Contributions

J-LW drafted the initial version of the manuscript and responded to the reviewers' comments. J-RS analyzed the data. H-ZW collected the data and provided the comments.

### Conflict of Interest Statement

The authors declare that the research was conducted in the absence of any commercial or financial relationships that could be construed as a potential conflict of interest.

## References

[1] Su YS Chiang WL Lee CTJ Chang HC The effect of flow experience on player loyalty in mobile game application. Comput Hum Behav. (2016) 63:240–8. 10.1016/j.chb.2016.05.049

[2] China Internet Network Information Center The 41th Statistical Report on the Development of Internet in China. (2018). Available online at: http://www.cnnic.net.cn/ (accessed October 30, 2018).

[3] Kandell JJ Internet addiction on campus: the vulnerability of college student. Cyber Psychol Behav. (1998) 1:11–8. 10.1089/cpb.1998.1.11

[4] Stockdale L Coyne SM. Video game addiction in emerging adulthood :cross-sectional evidence of pathology in video game addicts as compared to matched healthy controls. J Affect Disord. (2018) 225:265–72. 10.1016/j.jad.2017.08.045 28841491

[5] Sun Y Zhao Y Jia SQ Zheng DY Understanding the antecedents of mobile game addiction: the roles of perceived visibility, perceived enjoyment and flow. In: Proceedings of the 19th Pacific-Asia Conference on Information Systems. Singapore: Marian Bay Sands (2015). p. 1–12. Available online at: http://aisel.aisnet.org/pacis2015/141

[6] Sha P Sariyska R Riedl R Lachmann B Montag C. Linking Internet communication and smartphone use disorder by taking a closer look at the Facebook and WhatsApp applications. Addict Behav Rep. (2018) 9:100148. 10.1016/j.abrep.2018.100148 31193857 PMC6543448

[7] Lee C Kim O Predictors of online game addiction among Korean adolescents. Addict Res Theory. (2017) 25:58–66. 10.1080/16066359.2016.1198474

[8] Bozoglan B Demirer V Sahin I. Loneliness, self-esteem, and life satisfaction as predictors of Internet addiction: a cross-sectional study among Turkish university students. Scand J Psychol. (2013) 54:313–9. 10.1111/sjop.12049 23577670

[9] Ko C Yen J Chen C Yeh Y Yen C. Predictive values of psychiatric symptoms for Internet addiction in adolescents. JAMA Pediatrics. (2011) 163:937–43. 10.1001/archpediatrics.2009.159 19805713

[10] Taylor S (2017). The theoretical underpinnings of Internet addiction and its association with psychopathology in adolescence. Int J Adolesc Med Health. 2017:46 10.1515/ijamh-2017-0046 28682784

[11] Lawrence TL Peng Z-W Effect of pathological use of the Internet on adolescent mental health. JAMA Pediatrics. (2010) 164:901–6. 10.1001/archpediatrics.2010.159 20679157

[12] Mccauley C Video game play and anxiety during late adolescence: the moderating effects of gender and social context. J Affect Disord. (2018) 226:216–9. 10.1016/j.jad.2017.10.009 28992585 PMC5815171

[13] Liu L Yao YW Li CR Zhang JT Xia CC Lan JT. The comorbidity between internet gaming disorder and depression: interrelationship and neural mechanisms. Front Psychiatry. (2018) 9:154. 10.3389/fpsyt.2018.00154 29740358 PMC5924965

[14] Wu AM Chen JH Tong KK Yu S Lau JT. Prevalence and associated factors of Internet gaming disorder among community dwelling adults in Macao, China. J Behav Addict. (2018) 7:62–9. 10.1556/2006.7.2018.12 29463097 PMC6035015

[15] King DL Delfabbro PH King DL. The cognitive psychopathology of Internet gaming disorder in adolescence. J Abnormal Child Psychol. (2016) 44:1635–45. 10.1007/s10802-016-0135-y 26875565

[16] Blazer D Loneliness: a source book of current theory, research and therapy. J Behav Ther Exp Psychiatry. (1983) 14:281 10.1016/0005-7916(83)90066-6

[17] Spilkova J Chomynova P Csemy L. Predictors of excessive use of social media and excessive online gaming in Czech teenagers. J Behav Addict. (2017) 6:611–9. 10.1556/2006.6.2017.064 29039223 PMC6034940

[18] Van Rooij AJ Kuss DJ Griffiths MD Shorter GW Schoenmakers TM Van De Mheen D. The (co-) occurrence of problematic video gaming, substance use, and psychosocial problems in adolescents. J Behav Addict. (2014) 3:157–65. 10.1556/JBA.3.2014.013 25317339 PMC4189309

[19] Lemmens JS Valkenburg PM Peter J Development and validation of a game addiction scale for adolescents. Media Psychol. (2012) 12:77–95. 10.1080/15213260802669458

[20] Rapee RM Heimberg RG. A cognitive-behavioral model of anxiety in social phobia. Behav Res Ther. (1997) 35:741–56. 10.1016/S0005-7967(97)00022-3 9256517

[21] Maldonado L Huang Y Chen R Kasen S Cohen P Chen H. Impact of early adolescent anxiety disorders on self-esteem development from adolescence to young adulthood. J Adolesc Health. (2013) 53:287–92. 10.1016/j.jadohealth.2013.02.025 23648133 PMC3725205

[22] Merikangas KR He J Burstein M Swanson SA Avenevoli S Cui L. (2010). Lifetime prevalence of mental disorders in U.S. adolescents: results from the National Comorbidity Survey Replication-Adolescent Supplement (NCS-A). J Am Acad Child Adolesc Psychiatry. 49:980–9. 10.1016/j.jaac.2010.05.017 20855043 PMC2946114

[23] Fayazi M Hasani J Structural relations between brain-behavioral systems, social anxiety, depression and internet addiction: with regard to revised Reinforcement Sensitivity Theory (r-RST). Computers Human Behav. (2017) 72:441–8. 10.1016/j.chb.2017.02.068

[24] Lo S Wang C Fang W Physical interpersonal relationship and social anxiety among online game players. Cyber Psychol Behav. (2005) 8:15–20. 10.1089/cpb.2005.8.15 15738689

[25] Duven E Beutel ME Wolfling KJ (2013). The neuroscience of internet and computer game addiction—what do we know about what is going on inside our patients brains? Eur Psychiatry. 28:818 10.1016/S0924-9338(13)75997-2

[26] Yen C Chou W Liu T. The association of Internet addiction symptoms with anxiety, depression and self-esteem among adolescents with attention-deficit/hyperactivity disorder. Compr Psychiatry. (2014) 55:1601–8. 10.1016/j.comppsych.2014.05.025 25015304

[27] Shi X Wang J Zou H Family functioning and Internet addiction among Chinese adolescents: the mediating roles of self-esteem and loneliness. Computers Human Behav. (2017) 76:201–10. 10.1016/j.chb.2017.07.028

[28] Beranuy M Oberst U Carbonell X Chamarro A Problematic Internet and mobile phone use and clinical symptoms in college students: the role of emotional intelligence. Computers Human Behav. (2009) 25:1182–7. 10.1016/j.chb.2009.03.001

[29] Sheng J-R Wang J-L Development and psychometric properties of the problematic mobile video gaming scale. Curr Psyol. (2019) 20191–11. 10.1007/s12144-019-00415-6

[30] Derogatis LR Melisaratos N. The brief symptom inventory: an introductory report. Psychol Med. (1983) 13:595–605. 10.1017/S0033291700048017 6622612

[31] Li X Zou H Liu Y Psychometric evaluation of loneliness scale in Chinese middle school students. Chin J Clin Psychol. (2014) 22:731–60. 10.16128/j.cnki.1005-3611.2014.04.037

[32] La Greca AM Lopez N. Social anxiety among adolescents: linkages with peer relations and friendships. J Abnormal Child Psychol. (1998) 26:83–94. 10.1023/A:1022684520514 9634131

[33] Snedecor GW Cochran WG Statistical Methods, 8th ed. Ames, IA: Iowa State University Press (1989).

[34] Lim TS Loh WY A comparison of tests of equality of variances. Comput Stat Data Anal. (1996) 22:287–301. 10.1016/0167-9473(95)00054-2

[35] George D Mallery P SPSS for Windows Step by Step: A Simple Guide and Reference, 11.0 Update, 4th ed. Boston: Allyn & Bacon (2003).

[36] Johnson RA Wichern DW Applied Multivariate Statistical Analysis, 5th ed. Englewood Cliffs, NJ: Prentice Hall (2006).

[37] Harris J Hagger MS Do basic psychological needs moderate relationships within the theory of planned behavior? J Appl Biobehav Res. (2007) 12:43–64. 10.1111/j.1751-9861.2007.00013.x

[38] Saxton T Dollinger M Target reputation and appropriability: picking and deploying resources in acquisitions. J Manag. (2004) 30:123–47. 10.1016/j.jm.2003.01.006

[39] Wu ML Structural Equation Modeling: The Operation and Application of AMOS. Chongqing: Chongqing University Press (2009).

[40] Bruchas MR Schindler AG Shankar H Messinger DI Miyatake M Land BB Selective p38 a MAPK deletion in serotonergic neurons produces stress resilience in models of depression and addiction. Neuron. (2011) 71:498–511. 10.1016/j.neuron.2011.06.011 21835346 PMC3155685

[41] Whang LS Ph D Lee S Ph D Chang G. Internet over-users' psychological profiles: a behavior sampling analysis on internet addiction. Cyber Psychol Behav. (2003) 6:143–51. 10.1089/109493103321640338 12804026

[42] Yen J Ko C Yen C Chen S. Psychiatric symptoms in adolescents with Internet addiction : comparison with substance use. Psychiatry Clin Neurosci. (2008) 62:9–16. 10.1111/j.1440-1819.2007.01770.x 18289136

[43] Kraut R Patterson M Lundmark V. Internet paradox: a social technology that reduces social involvement and psychological well-being? Am Psychol. (1998) 53:1017–31. 10.1037//0003-066X.53.9.1017 9841579

[44] Wang J-L Jackson LA Zhang D-J The mediator role of self-disclosure and moderator roles of gender and social anxiety in the relationship between Chinese adolescents' online communication and their real-world social relationships. Computers Human Behav. (2011) 27:2161–8. 10.1016/j.chb.2011.06.010

[45] Schouten AP Valkenburg PM Peter J Precursors and underlying processes of adolescents' online self-disclosure: developing and testing an “internet-attribute-perception” model. Media Psychol. (2007) 10:292–315. 10.1080/15213260701375686

[46] Caplan SE. Relations Among Loneliness, Social Anxiety, and Problematic Internet Use. Cyber Psychol Behav. (2007) 10:234–42. 10.1089/cpb.2006.9963 17474841

